# Prehospital Stroke Care, Paramedic Training Needs, and Hospital-Directed Feedback in Lithuania

**DOI:** 10.3390/healthcare10101958

**Published:** 2022-10-07

**Authors:** Kazimieras Melaika, Lukas Sveikata, Aleksandras Vilionskis, Adam Wiśniewski, Kristaps Jurjans, Andrius Klimašauskas, Dalius Jatužis, Rytis Masiliūnas

**Affiliations:** 1 Center of Neurology, Vilnius University, 08661 Vilnius, Lithuania; 2 J. Philip Kistler Stroke Research Center, Department of Neurology, Massachusetts General Hospital, Harvard Medical School, Boston, MA 02114, USA; 3 Division of Neurology, Department of Clinical Neurosciences, Geneva University Hospitals, 1205 Geneva, Switzerland; 4 Institute of Cardiology, Medical Academy, Lithuanian University of Health Sciences, 50161 Kaunas, Lithuania; 5 Clinic of Neurology and Neurosurgery, Institute of Clinical Medicine, Vilnius University, 08661 Vilnius, Lithuania; 6 Department of Neurology, Collegium Medicum in Bydgoszcz, Nicolaus Copernicus University in Toruń, 85-067 Bydgoszcz, Poland; 7 Department of Neurology and Neurosurgery, Riga Stradins University, 1002 Riga, Latvia; 8 Department of Neurology, Pauls Stradins Clinical University Hospital, 1002 Riga, Latvia; 9 Center of Anaesthesiology, Intensive Therapy and Pain Management, Vilnius University, 08661 Vilnius, Lithuania

**Keywords:** survey, emergency medical services, training, stroke, prehospital care

## Abstract

Background: Emergency medical services (EMS) are the first health care contact for the majority of stroke patients. However, there is a lack of data on the current paramedics’ hospital-directed feedback and training needs across different health care settings. We aimed to evaluate paramedics’ prehospital stroke care knowledge, training needs, and current status of feedback on suspected stroke patients. Methods: We surveyed paramedics from the Vilnius region from September to November 2019 and compared the answers between the city and the district agencies. The questionnaire content included questions on paramedics’ demographic characteristics, prehospital stroke care self-assessment, knowledge on stroke mimics, stroke training needs, and the importance of hospital-directed feedback on suspected stroke patients. Results: A total number of 161 paramedics (or 49.4% of all paramedics from our stroke care network) were surveyed, with more district paramedics rating their prehospital stroke care knowledge as inadequate (44.8% (95% confidence interval (CI) 32.8–57.6) vs. 28.1% (95% CI 20.1–27.8), *p* = 0.028). In addition, more district paramedics indicated a need for additional stroke training (83.1% (95% CI 71.5–90.5) vs. 69.8% (60.0–78.1), *p* = 0.043). However, respondents reported being the most confident while dealing with stroke (71.3%, 95% CI 63.8–77.7) compared to other time-critical conditions (*p* < 0.001). Vertigo (60.8%, 95% CI 53.0–68.0), brain tumors (56.3%, 95% CI 48.5–63.8), and seizures (54.4%, 95% CI 46.7–62.0) were indicated as the most common stroke mimics. Only 6.2% (95% CI 3.4–11.1) of respondents received formal feedback on the outcome of suspected stroke patients brought to the emergency department. Conclusions: A high proportion of paramedics self-perceive having inadequate stroke knowledge and an urgent need for further stroke training. The EMS staff indicate receiving insufficient feedback on suspected stroke patients, even though its usefulness is perceived as paramount.

## 1. Introduction

Stroke is the second-leading cause of death and the third-leading cause of death and disability combined worldwide, and it is estimated to increase by 27% by 2047 [1,2]. It is a time-sensitive condition, as accurate recognition and timely transport of patients with suspected stroke to the nearest stroke-ready hospital is closely correlated with acute stroke care success [3,4]. Emergency Medical Services (EMS) play a crucial role in early stroke recognition as they are the first health care contact in about two-thirds of stroke patients [5]. Therefore, how paramedics respond to stroke is paramount in reducing prehospital delays and improving patient outcomes [6,7].

Accurate EMS dispatch, accurate stroke recognition, rapid transportation, and hospital stroke team prenotification substantially reduce prehospital patient delays and significantly increase reperfusion treatment rates, and, in parallel, improve the short-term and long-term patient outcomes [8]. However, correct identification of stroke patients in the prehospital care setting remains problematic, as EMS staff fail to identify up to one-third of stroke patients [9]. On the other hand, overdiagnosis of stroke (stroke mimics) might also cause a burden due to excessive load on the medical personnel, making them more prone to fatigue and burnout, as a result, lowering the timeliness and efficiency of the diagnostic and treatment processes in the emergency department. Thus, comprehensive interventions are crucial to increase the quality of prehospital care and reach the best patient-related outcomes.

One way to improve the quality of prehospital stroke care is through continuing professional development [10,11]. In addition, regular feedback to paramedics on their transported patients could also be an important tool that would enable learning from everyday practical experience [12,13]. However, data on the current paramedics’ hospital-directed feedback and training needs across different health care settings are lacking.

Therefore, we used a structured questionnaire to evaluate paramedics’ prehospital stroke care knowledge, training needs, and current status of feedback on suspected stroke outcomes. In addition, we compared urban and suburban EMS agencies, hypothesizing that the level of knowledge and access to medical training might differ based on the EMS location.

## 2. Materials and Methods

### 2.1. Study Design

We conducted a cross-sectional survey of Vilnius city and district paramedics between September and November 2019. A structured paper questionnaire was distributed to all the EMS staff who participated in stroke care training at Vilnius University Hospital [11] before the start of the training. To elucidate the factors associated with paramedics’ prehospital stroke care knowledge, training needs, and current status of feedback on suspected stroke outcomes, we reviewed the current literature on paramedic surveys concerning stroke care. We chose the questionnaire to be based on a survey of the United Kingdom’s (UK) paramedics, conducted by McClelland et al. [14], which aligned with our predefined research goals. A Lithuanian version of the survey was used, adapted to the Lithuanian prehospital setting. The questionnaire’s initial translation was evaluated by a committee from the Lithuanian Stroke Association. To take into account the comments received, a few minor changes were made. Then, a team of paramedics evaluated its readability. No modifications were made because the pilot version was deemed thorough and simple to finish. The content included questions on paramedics’ demographic characteristics, prehospital stroke care self-assessment, knowledge of stroke mimics, stroke training needs, the importance of hospital-directed feedback on suspected stroke patients, and attitudes toward the current Lithuanian stroke network. Respondents were asked to rate their confidence/influence/change using a 5-point Likert scale. The English translation of the Lithuanian version of the survey is presented as Supplementary Material S1.

### 2.2. Setting

Essential emergency health services in Lithuania are free of charge and EMS are the first responders in the majority of medical emergencies. Each EMS unit in Lithuania is staffed by a two-person team—a specialist-paramedic, and a driver-paramedic [11]. In urban areas, each EMS unit can serve up to a maximum of 18,000 inhabitants, and up to 16,000 inhabitants in suburban areas. Our study was conducted among Vilnius city and district paramedics, employed by eight EMS agencies—one operating in an urban and seven in suburban municipalities, covering a catchment population of approximately 945,000 inhabitants. Collectively, in 2019, these EMS agencies were staffed by 326 specialists (214 in urban and 112 in suburban locations) and transported ≈20,400 patients, of whom an estimated 5.0% were suspected strokes. Vilnius district stroke patients are carried to one of the two comprehensive stroke centers in Vilnius or a primary stroke center in Utena [15]. Regularly updated national Lithuanian stroke care guidelines encompass the prehospital stroke care setting [16]. Although following the National law, all EMS agencies across the country utilize identical dispatch protocols [17], and due to sheer geographic differences, the median onset-to-door time is significantly longer for stroke patients carried by suburban EMS agencies of our stroke care network, by around 44 min [11]. 

### 2.3. Ethics

Lithuanian legislation does not require ethical review or approval for anonymous surveys in which no personal data are collected. The study did not include any patients and the answers to the questionnaire were completely anonymized, no identifiable data were collected. Paramedics expressed their consent to participate in the study by voluntarily completing the anonymous survey form and they were able to revoke their consent and withdraw from further completion at any time.

### 2.4. Statistical Analysis

The data were reported descriptively with numerical and percentage frequencies. The χ2 test and Fisher’s exact test were used for categorical variables, as appropriate. Based on the EMS location, urban and suburban areas were compared. Respondents, working in both urban and suburban EMS agencies, were excluded from the comparison. *p* < 0.05 (two-sided) was considered statistically significant. IBM SPSS Statistics 23.0 software (Armonk, NY, USA: IBM Corp) and R version 3.6.2 were used for statistical analyses.

## 3. Results

### 3.1. Demographic Characteristics

In total, 176 participants attended the stroke care training, all of whom agreed to fill out the questionnaire. Fourteen of them were excluded from the analysis as they were working in a hospital but not in the EMS, and one respondent was an administrator of the EMS agency. Therefore, we included 161 out of 326 (49.4%) paramedics from our stroke care network (Table 1). Based on their EMS agency location, 97 (60.2%) paramedics worked in Vilnius city, 59 (36.6%) in Vilnius district, and 5 (3.2%)—in both urban and suburban agencies. The majority of the EMS staff were women (74.5%), and more than two-thirds of the respondents belonged to age groups between 40 and 59 years (mean age 49.9 ± 10.0 years). With only a small proportion of the paramedics below the age of 40 years (13.4%), the majority of the EMS staff (72.0%) indicated a long-term experience of 21 years or more working in prehospital care. The surveyed EMS employees indicated having a degree of a community nurse (83.8%), a paramedic (13.8%), or a medical doctor (2.5%). Despite their previous education, all participants included are referred to as paramedics, as defined by the College of Paramedics [18].

### 3.2. Stroke Care Knowledge and Training Needs

**Figure 1:**
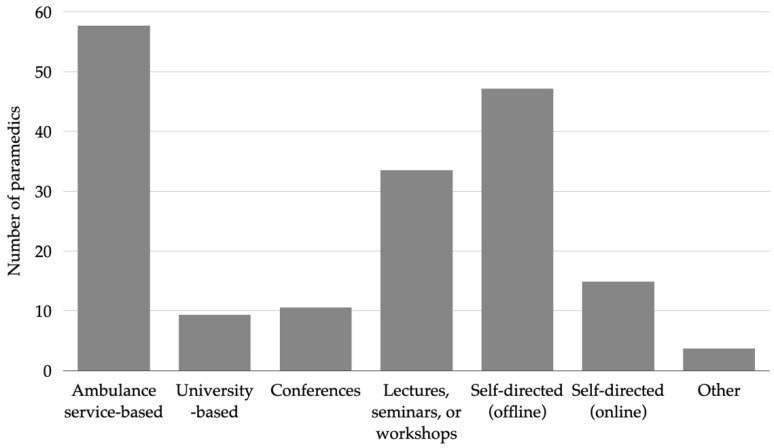
The sources where EMS respondents receive their primary stroke professional continuous education.

More district than city paramedics indicated having inadequate prehospital stroke care knowledge (44.8% (95% CI 32.8–57.6) vs. 28.1% (95% CI 20.1–37.8), *p* = 0.028) (Figure S1). The vast majority of the respondents (97.5%, 95% CI 93.8–99.0) indicated that they had improved their prehospital stroke knowledge since the beginning of their careers. The most common stroke professional continuous education source was ambulance service-based courses (57.8%, 95% CI 50.0–65.1), followed by self-directed offline sources (47.2%, 95% CI 39.7–54.9), and lectures, seminars, or workshops (33.5%, 95% CI 26.7–41.1) (Figure 1). The difference in current prehospital stroke care knowledge is consistent with further results—more suburban than urban paramedics indicated the need for further individual prehospital stroke care training (83.1% (95% CI 71.5–90.5) vs. 69.8% (95% CI 60.0–78.1), *p* = 0.043) (Figure S2). When asked about the need for continuous stroke training for EMS as a whole, high stroke training demand was indicated in city and district paramedics (85.1%, 95% CI 78.8–89.8).

### 3.3. Confidence, Influence, and Trends Dealing with Time-Critical Conditions

**Figure 2:**
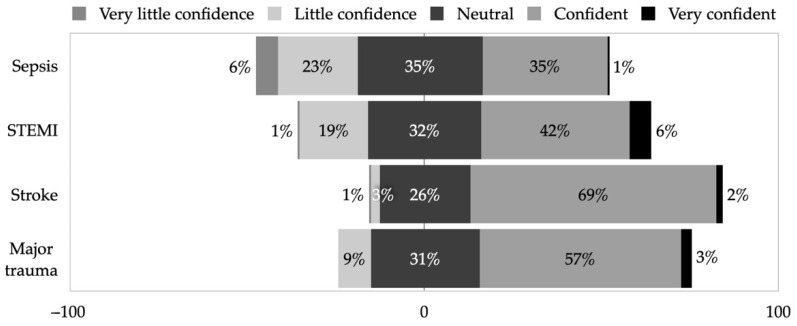
EMS staff’s evaluation of their confidence dealing with time-critical conditions.

First, paramedics rated their confidence in dealing with four different time-critical conditions, using a 5-point Likert scale (Figure 2). Although, paramedics were more confident while dealing with stroke (71.3% (95% CI 63.8–77.7), *p* < 0.001) compared to other conditions, almost one-third of respondents felt either neutral, or had little or very little confidence while dealing with suspected stroke. Accordingly, paramedics were less confident while dealing with major trauma (60.0%, 95% CI 52.3–67.3), ST-elevation myocardial infarction (STEMI) (48.1%, 95% CI 40.5–55.8), and sepsis (35.9%, 95% CI 28.8–43.6).

Then, the paramedics rated what influence prehospital care has on patient-related outcomes. The most influence was reported when dealing with STEMI (98.1%, 95% CI 94.7–99.4) and major trauma (97.5%, 95% CI 93.8–99.0), followed by stroke (91.3%, 95% CI 85.9–94.8) and sepsis (84.5%, 95% CI 78.1–89.3) (Figure S3). 

**Figure 3:**
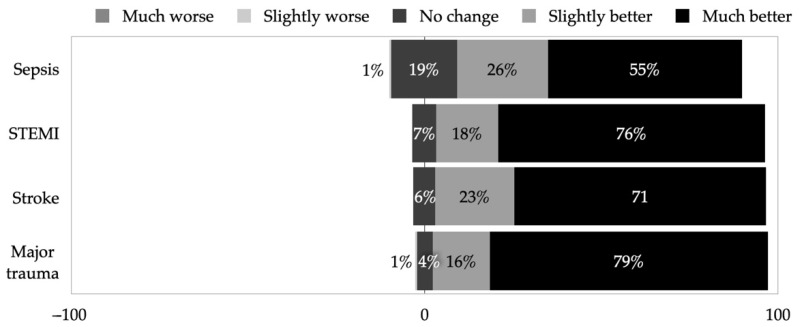
EMS staff evaluated the change of prehospital care quality over their career.

Lastly, the surveyed paramedics indicated that similar prehospital care improvement trends during their career were observed in all of the four time-critical conditions (Figure 3)—the most in major trauma (95.0%, 95% CI 90.4–97.4), stroke (93.8%, 95% CI 88.9–96.6), and STEMI (93.1%, 95% CI 88.1–96.1), and the least improvement was noted in sepsis care (80.6%). 

### 3.4. Conditions Mimicking Stroke

**Figure 4:**
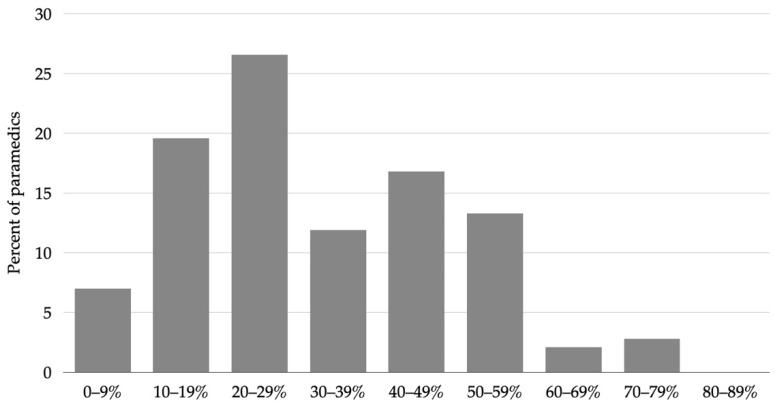
EMS estimate of stroke mimic prevalence in the prehospital setting.

Most paramedics (26.6%, 95% CI 20.0–34.4) estimated that the proportion of prehospital suspected stroke patients that were ultimately given a stroke mimic diagnosis ranged between 20 and 29 per cent (Figure 4). 

**Figure 5:**
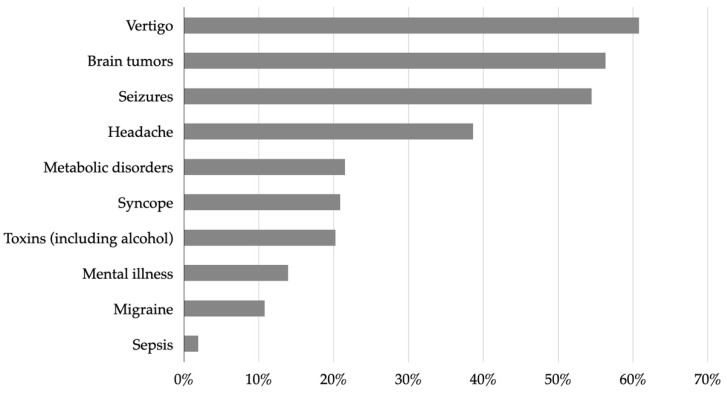
Conditions that were reported by the EMS staff as the most common stroke mimics.

EMS staff thought that the three most common stroke mimics were vertigo (60.8%, 95% CI 53.0–68.0), brain tumors (56.3%, 95% CI 48.5–63.8), and seizures (54.4%, 95% CI 46.7–62.0) (Figure 5). 

### 3.5. Feedback

Overall, 112 (70.4%, 95% CI 62.9–77.0) of the surveyed EMS staff agreed on the usefulness of hospital-directed feedback on suspected stroke outcomes (Figure S4). However, insufficient feedback was observed. Fifty-five (34.2%, 95% CI 27.3–41.8) paramedics reported not receiving any feedback at all, 96 (59.6%, 95% CI 51.9–66.9) noted occasionally receiving informal feedback, and only 10 (6.2%, 95% CI 3.4–11.1) indicated regularly receiving formal feedback. 

### 3.6. Attitude toward the Lithuanian Stroke Network

When asked about their attitude toward the current Lithuanian stroke care system, 30 (19.4%, 95% CI 13.9–26.3) of the respondents were very positive, 102 (65.8%, 95% CI 58.0–72.8)—positive, 18 (11.6%, 95% CI 7.5–17.6)—neutral, and only 5 (3.2%, 95% CI 1.4–7.3) expressed a negative or very negative view (Figure S5).

## 4. Discussion

This survey on prehospital stroke care, training needs, and hospital-directed feedback provided us with several main findings. First, we found a high proportion of EMS specialists who indicated having inadequate stroke knowledge and high stroke training needs, significantly more prominent in suburban agencies. Second, paramedics felt more confident dealing with stroke than with other time-critical conditions despite the inadequate self-perceived stroke knowledge. Third, we found that paramedics receive an insufficient amount of hospital-directed feedback on suspected stroke patients, even though the usefulness of such feedback was considered of high significance. We discuss the possible reasons and implications below.

Our survey revealed that the absolute majority of the EMS staff have continued to improve their prehospital stroke care skills since the beginning of their careers. However, only a third of respondents marked having participated in lectures, seminars, or workshops and only one in ten paramedics participated in university-based curriculum and conferences. Similar stroke continuing professional development proportions were found in a UK paramedics’ survey, although involvement in any continuing professional development training was at least twice as high [14].

There are no known differences in continuous education in urban and suburban regions in our study as all EMS agencies included in the study receive their continuous education in a centralized manner. However, significantly more suburban paramedics rated their prehospital stroke care knowledge as inadequate and indicated significantly higher stroke training demand for EMS as a whole, although an overwhelming training demand was observed in both groups. These findings are in line with other studies, showing that EMS staff members lacked stroke care knowledge [8,14,19], and regional disparities were present [20]. Studies assessing the real-world impact of EMS training show that it improves stroke recognition, increases hospital prenotification rates, improves tPA delivery time [10,11], and stroke transfer time to the ED [3]. As emphasized by the European [3] and North American guidelines [21], it is crucial to maintain the continuity of EMS education. This is especially important in response to ever-changing external factors, such as global public health emergencies [5,22] or changes in the standard operating procedures due to the advent of telemedicine and Mobile Stroke Units [23,24,25,26]. The shifting landscape of prehospital stroke care puts the spotlight on EMS training to improve prehospital stroke care competencies.

Despite the high demand for stroke training, paramedics expressed being most confident in dealing with stroke among other time-critical conditions. In comparison, a similar survey revealed that the UK paramedics were most confident in contact with sepsis, STEMI, and stroke patients, and the least confident when dealing with major trauma [14]. These differences might occur due to different exposure to certain conditions in prehospital care and varying availability of evidence-based guidelines. However, this could also indicate that EMS staff members might lack sufficient training or evidence-based guidelines for other time-critical conditions, despite the fact that responsibilities in the prehospital care field will continue to grow, therefore, continuous EMS retraining will be crucial [20,27]. In comparison, the UK paramedics expressed the least influence, and improvement in prehospital care when dealing with stroke [14]. 

Our survey revealed that only 6.2% of paramedics receive formal feedback on stroke, however, more than two-thirds of them think that hospital-directed feedback is crucial and would improve their future decision-making. These findings are in line with other studies, where formal feedback is noted as highly desirable for EMS staff members, yet not sufficient [8,10,12,13,14,19,28,29,30]. Previous studies have shown that hospital-directed feedback to EMS is associated with improved overall compliance with state protocols, increased hospital prenotification, and improved stroke timeliness metrics in patients with ischemic stroke [12]. 

Different legal patient data protection frameworks, such as the General Data Protection Regulation—the principal legal framework that regulates the collection and use of personal data within the European Union—could be one of the legal obstacles to efficient hospital-directed feedback. Nevertheless, previous position statements on process and outcomes data sharing between EMS and receiving hospitals offer recommendations for how the barriers to bilateral information exchange could be resolved [31], e.g., routinely provide hospital discharge summaries of patients transported to the ED. To avoid violating regulations on patient data protection, a United States-based EMS Management Association position statement recommends a healthcare institution employ a Privacy Officer to review complicated scenarios and regulations regarding information exchange, creating regulation-compliant standard operating procedures [31]. Three specific factors must be met to share protected health information on a given patient: (1) both the hospital and the ambulance service must have a patient relationship, (2) information must be pertinent to the parties, and (3) the disclosing party must release the “minimum information necessary”.

Most of the surveyed paramedics estimated that the proportion of stroke mimics ranges from 20 to 29 per cent, consistent with the real-world situation [5,10,32]. The most common stroke mimics indicated by the paramedics were vertigo, brain tumors, and seizures. We acknowledge the heterogeneity of stroke presentation, and in line with this, our stroke training program aimed to improve the paramedics’ stroke knowledge, raise awareness of stroke mimics, and increase the accuracy of stroke identification. Indeed, a previous study found seizures, vertigo, and hypertensive encephalopathy to be the three most common stroke mimics, with brain tumors being the seventh most common mimic [5]. Although similar trends have been observed in other studies, common stroke mimics may differ depending on a specific health care setting. For example, studies in the UK indicate seizure, migraine, sepsis, and syncope being the most common stroke mimics [8,14], whereas a Norwegian study noted infection, seizures, and dizziness or vertigo [33]. This highlights an enormous spectrum of diseases to be considered for the differential diagnosis of stroke, further emphasizing the important role that continuous professional development plays in improving prehospital stroke care [5].

Lastly, the current stroke network was recognized positively by almost 9 out of 10 EMS staff members. Previous studies have shown that a Lithuanian comprehensive national stroke care policy has resulted in significant trend improvements in reperfusion treatment rates, and a sustained significant decreasing trend of all-cause in-hospital case-fatality rates in stroke centers [15]. Future studies should aim to evaluate prehospital stroke care performance measures on the national level to investigate if the subjective positive assessment is based on stroke outcome improvements.

The main strength of our study was that, to our knowledge, this is the first survey in Eastern Europe that targeted the prehospital stroke care aspects. As Eastern Europe belongs to a very high cardiovascular risk region [34], there is an urgent need to study ways of improving the time-sensitive prehospital stroke care. Given that EMS staffing models and dispatch systems bear resemblance to other countries in the region [35,36], the results could be of considerable value in assessing the regional situation.

The main limitation of our study was that only half of the Lithuanian EMS agencies were involved in the survey. However, both urban and rural paramedic agencies have been represented, national regulations are imposed throughout the country, and all paramedics, participating in the EMS training, agreed to complete the survey. Thus, the risk of sampling and confirmation bias was minimal. Finally, the questionnaire was translated and adapted for the Lithuanian prehospital setting without a prior validation study. Although inherent to limitations, the questionnaire was used as a cross-sectional screen of the current EMS knowledge, and therefore we did not run into the risk of limited comparability. Future studies should validate a questionnaire to monitor the longitudinal change of EMS knowledge.

## 5. Conclusions

Our survey revealed that a high proportion of Lithuanian EMS specialists indicate having inadequate stroke knowledge and high stroke training needs, significantly more prominent in suburban agencies. Despite the inadequate self-perceived stroke knowledge, paramedics feel more confident dealing with stroke compared with other time-critical conditions. Finally, the paramedics receive an insufficient amount of feedback on suspected stroke patients, even though the usefulness is perceived to be paramount.

## Figures and Tables

**Table 1:** Paramedics’ demographic characteristics (n = 161).

	n (%)	
Female gender	120	(74.5)
Age range (years)		
20–29	10	(6.4)
30–39	11	(7.0)
40–49	53	(33.8)
50–59	58	(36.9)
60–69	24	(15.3)
70+	1	(0.6)
Experience in prehospital care (years)		
<2	6	(3.7)
3–5	14	(8.7)
6–10	8	(5.0)
11–20	17	(10.6)
21–30	58	(36.0)
31–40	44	(27.3)
41+	14	(8.7)
Ambulance employer		
Vilnius city	97	(60.2)
Vilnius district	59	(36.6)
Both	5	(3.2)
Education		
Community nurse	134	(83.8)
Paramedic	22	(13.8)
Medical doctor	4	(2.5)

## Data Availability

The data that support the findings of this study are available from the corresponding author upon reasonable request.

## Supplementary Materials

The following supporting information can be downloaded at: https://www.mdpi.com/article/10.3390/healthcare10101958/s1, Supplementary Material S1: Questionnaire; Figure S1: EMS self-reported prehospital stroke care knowledge in Vilnius city and districts; Figure S2: EMS self-reported need for continuous professional development in Vilnius city and districts; Figure S3: EMS staff’s evaluation of how prehospital care impacts patient-related outcomes; Figure S4: EMS received feedback on stroke patients; Figure S5: EMS attitude on current Lithuanian stroke care system.

## References

[1] Collaborators G.S., Feigin V.L., Stark B.A., Johnson C.O., Roth G.A., Bisignano C., Abady G.G., Abbasifard M., Abbasi-Kangevari M., Abd-Allah F. (2021). Global, Regional, and National Burden of Stroke and Its Risk Factors, 1990–2019: A Systematic Analysis for the Global Burden of Disease Study 2019. Lancet Neurol..

[2] Wafa H.A., Wolfe C.D.A., Emmett E., Roth G.A., Johnson C.O., Wang Y. (2020). Burden of Stroke in Europe: Thirty-Year Projections of Incidence, Prevalence, Deaths, and Disability-Adjusted Life Years. Stroke.

[3] Kobayashi A., Czlonkowska A., Ford G.A., Fonseca A.C., Luijckx G.J., Korv J., Ossa N.P., Price C., Russell D., Tsiskaridze A. (2018). European Academy of Neurology and European Stroke Organization Consensus Statement and Practical Guidance for Pre-hospital Management of Stroke. Eur. J. Neurol..

[4] Emberson J., Lees K.R., Lyden P., Blackwell L., Albers G., Bluhmki E., Brott T., Cohen G., Davis S., Donnan G. (2014). Effect of Treatment Delay, Age, and Stroke Severity on the Effects of Intravenous Thrombolysis with Alteplase for Acute Ischaemic Stroke: A Meta-Analysis of Individual Patient Data from Randomised Trials. Lancet.

[5] Melaika K., Sveikata L., Wiśniewski A., Jaxybayeva A., Ekkert A., Jatužis D., Masiliūnas R. (2021). Changes in Prehospital Stroke Care and Stroke Mimic Patterns during the COVID-19 Lockdown. Int. J. Environ. Res. Public Health.

[6] Mueller-Kronast N., Froehler M.T., Jahan R., Zaidat O., Liebeskind D., Saver J.L., Investigators S. (2020). Impact of EMS Bypass to Endovascular Capable Hospitals: Geospatial Modeling Analysis of the US STRATIS Registry. J. Neurointerv. Surg..

[7] Zhang S., Zhang J., Zhang M., Zhong G., Chen Z., Lin L., Lou M. (2018). Prehospital Notification Procedure Improves Stroke Outcome by Shortening Onset to Needle Time in Chinese Urban Area. Aging Dis..

[8] Li T., Munder S.P., Chaudhry A., Madan R., Gribko M., Arora R. (2019). Emergency Medical Services Providers’ Knowledge, Practices, and Barriers to Stroke Management. Open Access Emerg. Med..

[9] Abboud M.E., Band R., Jia J., Pajerowski W., David G., Guo M., Mechem C.C., Messé S.R., Carr B.G., Mullen M.T. (2016). Recognition of Stroke by EMS Is Associated with Improvement in Emergency Department Quality Measures. Prehospital Emerg. Care.

[10] Oostema J.A., Chassee T., Baer W., Edberg A., Reeves M.J. (2019). Brief Educational Intervention Improves Emergency Medical Services Stroke Recognition. Stroke.

[11] Sveikata L., Melaika K., Wiśniewski A., Vilionskis A., Petrikonis K., Stankevičius E., Jurjans K., Ekkert A., Jatužis D., Masiliūnas R. (2022). Interactive Training of the Emergency Medical Services Improved Prehospital Stroke Recognition and Transport Time. Front. Neurol..

[12] Choi B., Tsai D., McGillivray C.G., Amedee C., Sarafin J.-A., Silver B. (2018). Hospital-Directed Feedback to Emergency Medical Services Improves Prehospital Performance. Stroke.

[13] Eaton-Williams P., Mold F., Magnusson C. (2020). Effective Clinical Feedback Provision to Ambulance Clinicians: A Literature Review. J. Paramed. Pract..

[14] McClelland G., Flynn D., Rodgers H., Price C. (2017). A Survey of UK Paramedics’ Views about Their Stroke Training, Current Practice and the Identification of Stroke Mimics. Br. Paramed. J..

[15] Masiliūnas R., Vilionskis A., Bornstein N.M., Rastenytė D., Jatužis D. (2022). The Impact of a Comprehensive National Policy on Improving Acute Stroke Patient Care in Lithuania. Eur. Stroke J..

[16] Jatužis D., Rastenytė D., Vilionskis A., Matijošaitis V., Ryliškienė K. (2021). Galvos Smegenų Insulto Diagnostikos, Gydymo Ir Profilaktikos Metodika.

[17] Ministry of Health of the Republic of Lithuania. https://e-seimas.lrs.lt/portal/legalAct/lt/TAD/TAIS.437212/asr.

[18] Eaton G. (2019). Paramedic. Noun. Br. Paramed. J..

[19] Brunton L., Boaden R., Knowles S., Ashton C., Parry-Jones A.R. (2019). Pre-Hospital Stroke Recognition in a UK Centralised Stroke System: A Qualitative Evaluation of Current Practice. Br. Paramed. J..

[20] Williams I., Valderrama A.L., Bolton P., Greek A., Greer S., Patterson D.G., Zhang Z. (2011). Factors Associated with Emergency Medical Services Scope of Practice for Acute Cardiovascular Events. Prehospital Emerg. Care.

[21] Powers W.J., Rabinstein A.A., Ackerson T., Adeoye O.M., Bambakidis N.C., Becker K., Biller J., Brown M., Demaerschalk B.M., Hoh B. (2019). Guidelines for the Early Management of Patients with Acute Ischemic Stroke: 2019 Update to the 2018 Guidelines for the Early Management of Acute Ischemic Stroke: A Guideline for Healthcare Professionals from the American Heart Association/American Stroke Association. Stroke.

[22] Suppan M., Stuby L., Carrera E., Cottet P., Koka A., Assal F., Savoldelli G.L., Suppan L. (2021). Asynchronous Distance Learning of the National Institutes of Health Stroke Scale During the COVID-19 Pandemic (E-Learning vs. Video): Randomized Controlled Trial. J. Med. Internet Res..

[23] Grotta J.C., Yamal J.-M., Parker S.A., Rajan S.S., Gonzales N.R., Jones W.J., Alexandrov A.W., Navi B.B., Nour M., Spokoyny I. (2021). Prospective, Multicenter, Controlled Trial of Mobile Stroke Units. N. Engl. J. Med..

[24] Ebinger M., Siegerink B., Kunz A., Wendt M., Weber J.E., Schwabauer E., Geisler F., Freitag E., Lange J., Behrens J. (2021). Association Between Dispatch of Mobile Stroke Units and Functional Outcomes Among Patients with Acute Ischemic Stroke in Berlin. JAMA.

[25] Ramanathan R.S., Wisco D., Vela-Duarte D., Zafar A., Taqui A., Winners S., Buletko A.B., Hustey F., Reimer A., Russman A. (2021). Pre-Hospital Diagnosis in Mobile Stroke Unit. J. Stroke Cerebrovasc. Dis..

[26] Phillips D., Grunwald I.Q., Walter S., Faßbender K. (2021). Mobile Stroke Unit in the UK Healthcare System: Avoidance of Unnecessary Accident and Emergency Admissions. Br. Paramed. J..

[27] Koivulahti O., Tommila M., Haavisto E. (2020). The Accuracy of Preliminary Diagnoses Made by Paramedics—A Cross-Sectional Comparative Study. Scand. J. Trauma Resusc. Emerg. Med..

[28] Pollard J., Black S. (2015). Do paramedics find it beneficial to learn the diagnosis given to their patients in the emergency department?. Emerg. Med. J..

[29] Cash R.E., Crowe R.P., Rodriguez S.A., Panchal A.R. (2017). Disparities in Feedback Provision to Emergency Medical Services Professionals. Prehospital Emerg. Care.

[30] McGuire S.S., Luke A., Klassen A.B., Myers L.A., Mullan A.F., Sztajnkrycer M.D. (2021). It’s Time to Talk to Prehospital Providers: Feedback Disparities among Ground-Based Emergency Medical Services Providers and Its Impact on Job Satisfaction. Prehospital Disaster Med..

[31] Gunderson M.R., Florin A., Price M., Reed J. (2020). NEMSMA Position Statement and White Paper: Process and Outcomes Data Sharing between EMS and Receiving Hospitals. Prehospital Emerg. Care.

[32] Gibson L., Whiteley W. (2013). The Differential Diagnosis of Suspected Stroke: A Systematic Review. J. R. Coll. Physicians Edinb..

[33] Khanevski A.N., Kvistad C.E., Novotny V., Næss H., Thomassen L., Logallo N., Bjerkreim A.T. (2019). Incidence and Etiologies of Stroke Mimics after Incident Stroke or Transient Ischemic Attack. Stroke.

[34] Visseren F.L.J., Mach F., Smulders Y.M., Carballo D., Koskinas K.C., Bäck M., Benetos A., Biffi A., Boavida J.-M., Capodanno D. (2022). 2021 ESC Guidelines on Cardiovascular Disease Prevention in Clinical Practice. Eur. J. Prev. Cardiol..

[35] Karliński M., Gluszkiewicz M., Członkowska A. (2015). The Accuracy of Prehospital Diagnosis of Acute Cerebrovascular Accidents: An Observational Study. Arch. Med. Sci. Ams..

[36] Kozera-Strzelińska D., Karliński M., Rak G., Wojdacz M., Sienkiewicz-Jarosz H., Kurkowska-Jastrzębska I. (2019). Stroke and TIA Mimics in Patients Referred to a Neurological Emergency Department by Non-Ambulance Physicians, Ambulance Physicians and Paramedics. Neurol. Neurochir. Pol..

