# Supplementary material for: Prehospital Stroke Care, Paramedic Training Needs, and Hospital-Directed Feedback in Lithuania
Source: Healthcare (Basel). 2022 Oct 7;10(10):1958. doi: 10.3390/healthcare10101958 (PMC9601945; doi:10.3390/healthcare10101958)
Supplement: Supplementary file 1 [file healthcare-10-01958-s001.zip › Supplementary Material S1.pdf]

Supplementary material S1.

Stroke Care, Paramedic Training Needs, and Hospital-Directed Feedback in Lithuania

The survey is anonymous and all collected data will be used for scientific research purposes only.

We look forward to your sincere and complete answers.

QUESTIONNAIRE (mark the most appropriate answers)

Q1. WHAT IS YOUR GENDER?

1. Female
2. Male

Q2. WHAT IS YOUR AGE? ________ years.

Q3. WHICH SERVICE DO YOU WORK FOR?

1. Vilnius city EMS
2. Vilnius district EMS
3. Other (please specify) ______________________________________

Q4. WHAT IS YOUR HIGHEST LEVEL OF EDUCATION RELEVANT TO YOUR PARAMEDIC ROLE?

1. Community nurse
2. Paramedic
3. Medical doctor
4. Other (please specify) ______________________________________

Q5. HOW LONG HAVE YOU BEEN WORKING IN PREHOSPITAL CARE? ___________________ years.

Q6. BASED ON YOUR CURRENT KNOWLEDGE OF STROKE CARE HOW WOULD YOU CLASSIFY YOUR INITIAL TRAINING?

1. Inadequate
2. Adequate
3. Excessive

Q7. WOULD YOU LIKE MORE TRAINING ON PREHOSPITAL STROKE CARE?

1. Yes
2. No
3. Unsure

Q8. DO YOU THINK PARAMEDICS AS A GROUP NEED MORE TRAINING ON PREHOSPITAL STROKE CARE?

1. Yes
2. No
3. Unsure

Q9. WHAT TYPE OF TRAINING WOULD YOU LIKE REGARDING STROKE GOING FORWARD?

____

____

____

____

Q10. WHAT TYPE OF STROKE CONTINUING PROFESSIONAL DEVELOPMENT (CPD) ACTIVITIES FOCUSSED ON STROKE HAVE YOU COMPLETED SINCE QUALIFYING AS A PARAMEDIC?

1. Ambulance service based training
2. University based training
3. Conference with stroke related content
4. Stroke specific lecture, seminar or workshop
5. Self directed learning (offline)
6. Self directed learning (online)
7. Other (please specify) ____

Q11. WHAT STROKE ASSESSMENT TOOLS HAVE YOU BEEN TRAINED TO USE?

1. Cincinnati Prehospital Stroke Scale (CPSS)
2. Face Arms Speech Time (FAST)
3. Los Angeles Motor Scale (LAMS)
4. Los Angeles Prehospital Stroke Screen (LAPSS)
5. Melbourne Ambulance Stroke Screen (MASS)
6. Miami Emergency Neurologic Deficit (MEND)
7. National Institutes of Health Stroke Scale (NIHSS)
8. Rapid Arterial oCclusion Evaluation (RACE)
9. Recognition of Stroke in the Emergency Room (ROSIER)
10. Other (please specify) ____

Q12. HOW WOULD YOU RATE YOUR CONFIDENCE WHEN DEALING WITH THE FOLLOWING TIME CRITICAL CONDITIONS?

A. STROKE

1. Very little confidence
2. Little confidence
3. Neutral
4. Confident
5. Very confident

B. SEPSIS

1. Very little confidence
2. Little confidence
3. Neutral
4. Confident
5. Very confident

C. MAJOR TRAUMA

1. Very little confidence
2. Little confidence
3. Neutral
4. Confident
5. Very confident

D. STEMI

1. Very little confidence
2. Little confidence
3. Neutral
4. Confident
5. Very confident

Q13. TO WHAT EXTENT DO YOU THINK THAT PREHOSPITAL ACTIONS INFLUENCE A PATIENT‘S OUTCOME IN THE FOLLOWING TIME CRITICAL CONDITIONS?

A. STROKE

1. Very little influence
2. Little influence
3. Neutral
4. Some influence
5. Very high influence

B. SEPSIS

1. Very little influence
2. Little influence
3. Neutral
4. Some influence
5. Very high influence

C. MAJOR TRAUMA

1. Very little influence
2. Little influence
3. Neutral
4. Some influence
5. Very high influence

D. STEMI

1. Very little influence
2. Little influence
3. Neutral
4. Some influence
5. Very high influence

Q14. HOW DO YOU THINK PREHOSPITAL CARE HAS CHANGED OVER THE COURSE OF YOUR CAREER FOR THE FOLLOWING TIME CRITICAL CONDITIONS?

A. STROKE

1. Much worse
2. Slightly worse
3. No change
4. Slightly better
5. Much better

B. SEPSIS

1. Much worse
2. Slightly worse
3. No change
4. Slightly better
5. Much better

C. MAJOR TRAUMA

1. Much worse
2. Slightly worse
3. No change
4. Slightly better
5. Much better

D. STEMI

1. Much worse
2. Slightly worse
3. No change
4. Slightly better
5. Much better

Q15. DO YOU GET ANY FEEDBACK ON STROKE PATIENTS YOU TREAT AND TRANSPORT?

1. Yes (formal)
2. Sometimes (informal)
3. No

Q16. DO YOU THINK THAT FEEDBACK ON STROKE PATIENTS YOU TREAT WOULD BE USEFUL TO YOU AS A PARAMEDIC?

1. Yes
2. No (if you have answered no, go to Q19)
3. Unsure

Q17. HOW WOULD YOU LIKE THE FEEDBACK AND TO WHAT LEVEL OF DETAIL?

____
____
____
____

Q18. HOW WOULD YOU LIKE TO RECEIVE THE FEEDBACK?

1. By telephone from the doctor that admits the patient
2. Depersonalized via email
3. During EMS morning meetings
4. Other (please specify) ____
   ____
   ____

Q19. WOULD YOU ROUTINELY PERFORM AN ECG ON A STROKE PATIENT?

1. Yes – all stroke patients
2. Yes – only if they had chest pain
3. Not routinely

Q20. HOW LONG AFTER THE LAST KNOWN WELL TIME DO YOU THINK INTRAVENOUS THROMBOLYSIS CAN BE PERFORMED IN ACUTE ISCHEMIC STROKE PATIENTS?

1. <2.5 hours
2. <3.5 hours
3. <4.5 hours
4. <5.5 hours

A stroke mimic is a condition which presents like a stroke, but is actually due to different underlying pathology. Examples of stroke mimics would be the hypoglycaemic patient who presents with unilateral weakness and slurred speech or the patient with sudden onset facial weakness due to Bell's Palsy.

Q21. WHAT PROPORTION OF ALL PATIENTS ADMITTED BY AMBULANCE DO YOU THINK ARE ACUTE STROKE? (PLEASE SPECIFY) _____ %.

Q22. WHAT PROPORTION OF SUSPECTED STROKES ADMITTED BY AMBULANCE DO YOU THINK ARE STROKE MIMICS?

1. 0-9%.
2. 10-19%
3. 20-29%
4. 30-39%
5. 40-49%
6. 50-59%
7. 60-69%
8. 70-79%
9. 80-89%
10. 90-99%

Q23. WHAT DO YOU THINK ARE THE **THREE MOST COMMON** STROKE MIMICS SEEN IN PREHOSPITAL CARE?

1. Mental illness
2. Metabolic disorders
3. Migraine
4. Vertigo
5. Syncope
6. Headache
7. Brain tumours
8. Toxins (including alcohol)
9. Seizures
10. Sepsis

Q24. WHAT IS YOUR ATTITUDE REGARDING THE CURRENT LITHUANIAN STROKE CARE NETWORK, REFORMED IN 2014?

1. Very negative
2. Negative
3. Neither positive nor negative
4. Positive
5. Very positive

Q25. WHAT ARE THE FACTORS THAT DETERMINES YOUR CHOICE TO WHICH HOSPITAL TO TAKE A PATIENT WITH A SUSPECTED STROKE?

____
____
____
____

Q26. FROM YOUR EXPERIENCE, DO YOU HAVE ANY SUGGESTIONS HOW CAN LITHUANIA‘S STROKE ASSOCIATION AND NEUROLOGISTS COULD IMPROVE CARE FOR STROKE PATIENTS IN THE FUTURE?

____
____
____
____

THANK YOU FOR YOUR SINCERE ANSWERS
