# Supplementary figures and images for: Prehospital Stroke Care, Paramedic Training Needs, and Hospital-Directed Feedback in Lithuania
Source: Healthcare (Basel). 2022 Oct 7;10(10):1958. doi: 10.3390/healthcare10101958 (PMC9601945; doi:10.3390/healthcare10101958)

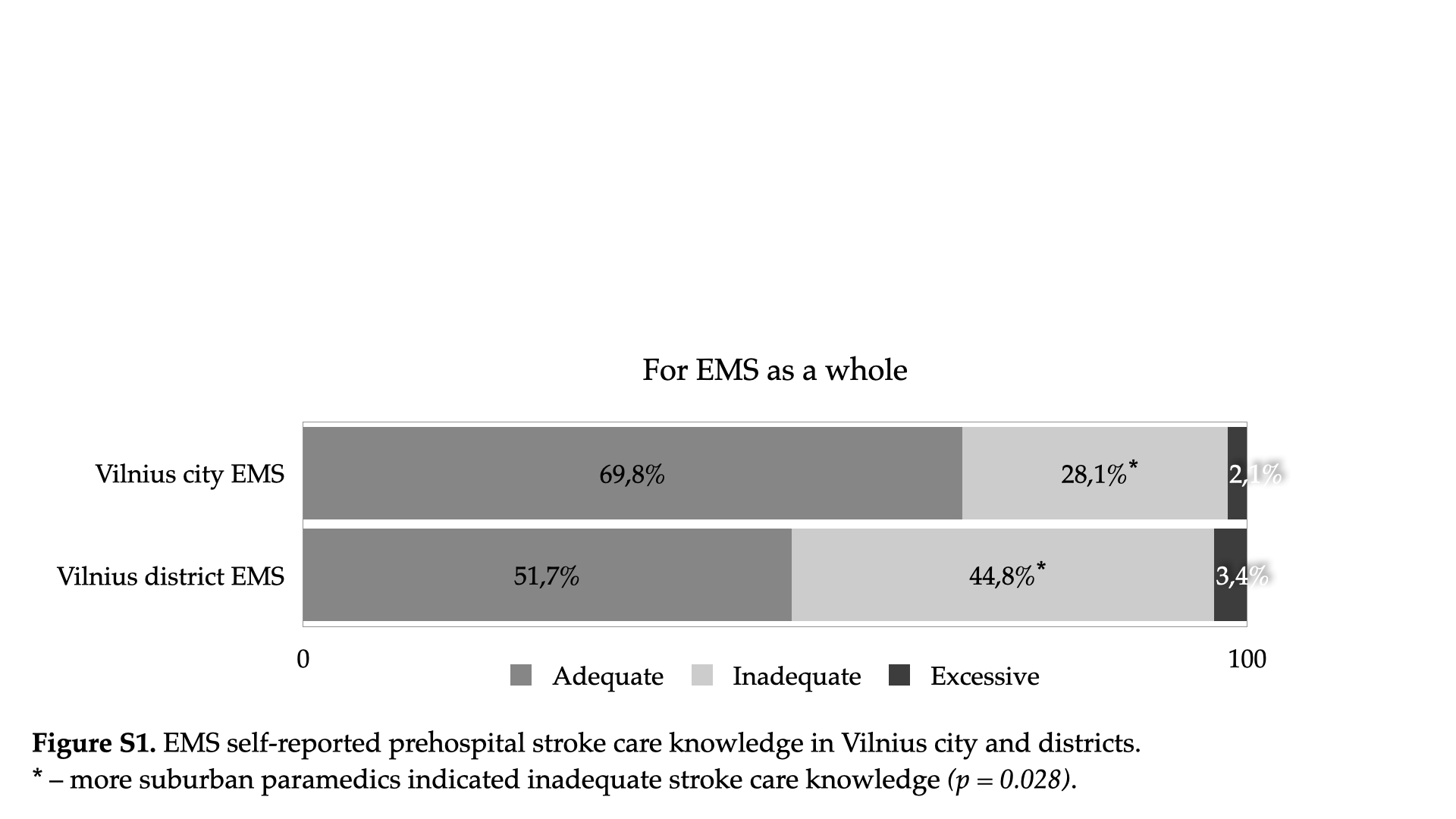

Supplement: Supplementary file 1 [file healthcare-10-01958-s001.zip › Figure S1.jpeg]

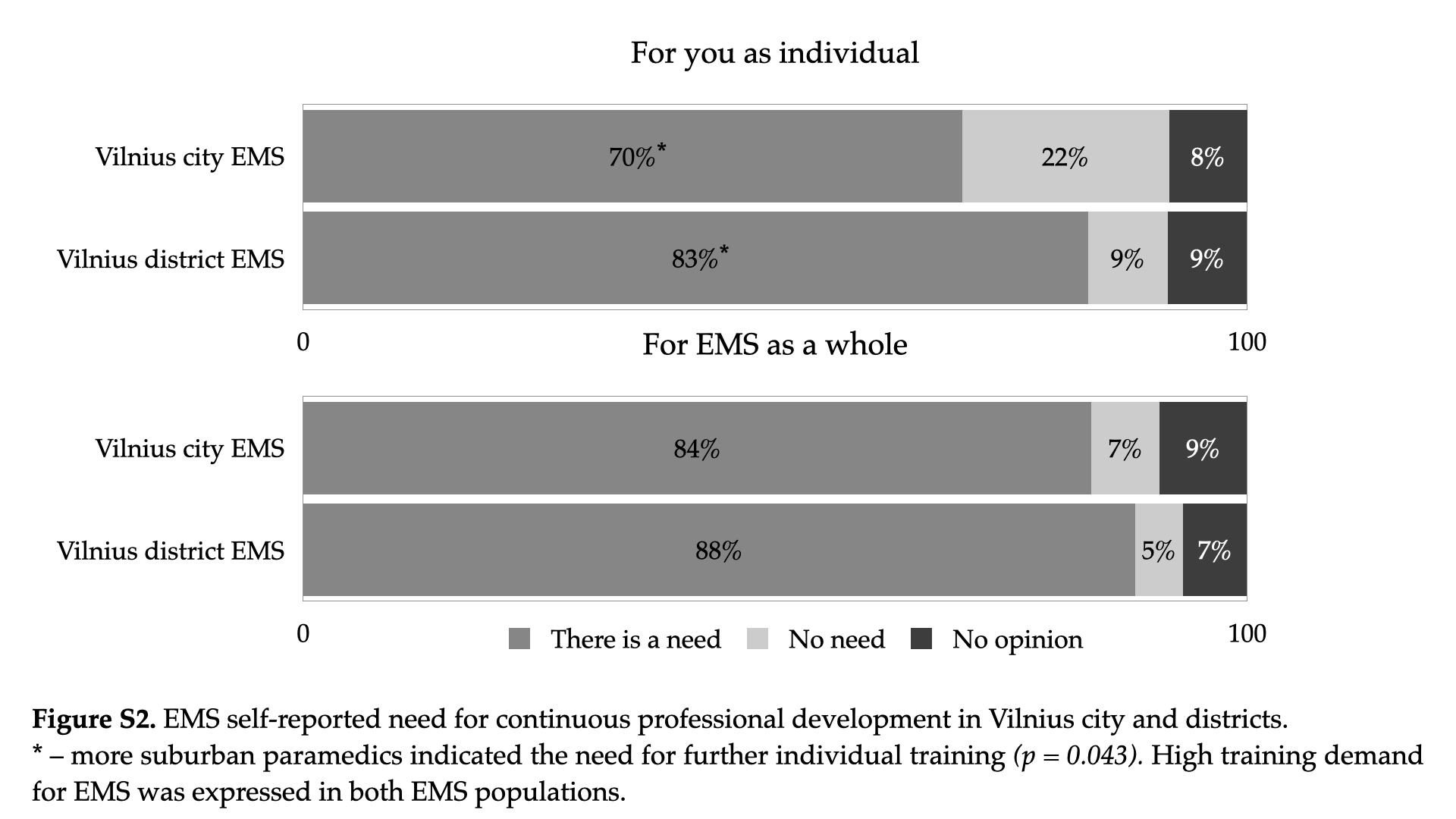

Supplement: Supplementary file 1 [file healthcare-10-01958-s001.zip › Figure S2.jpeg]

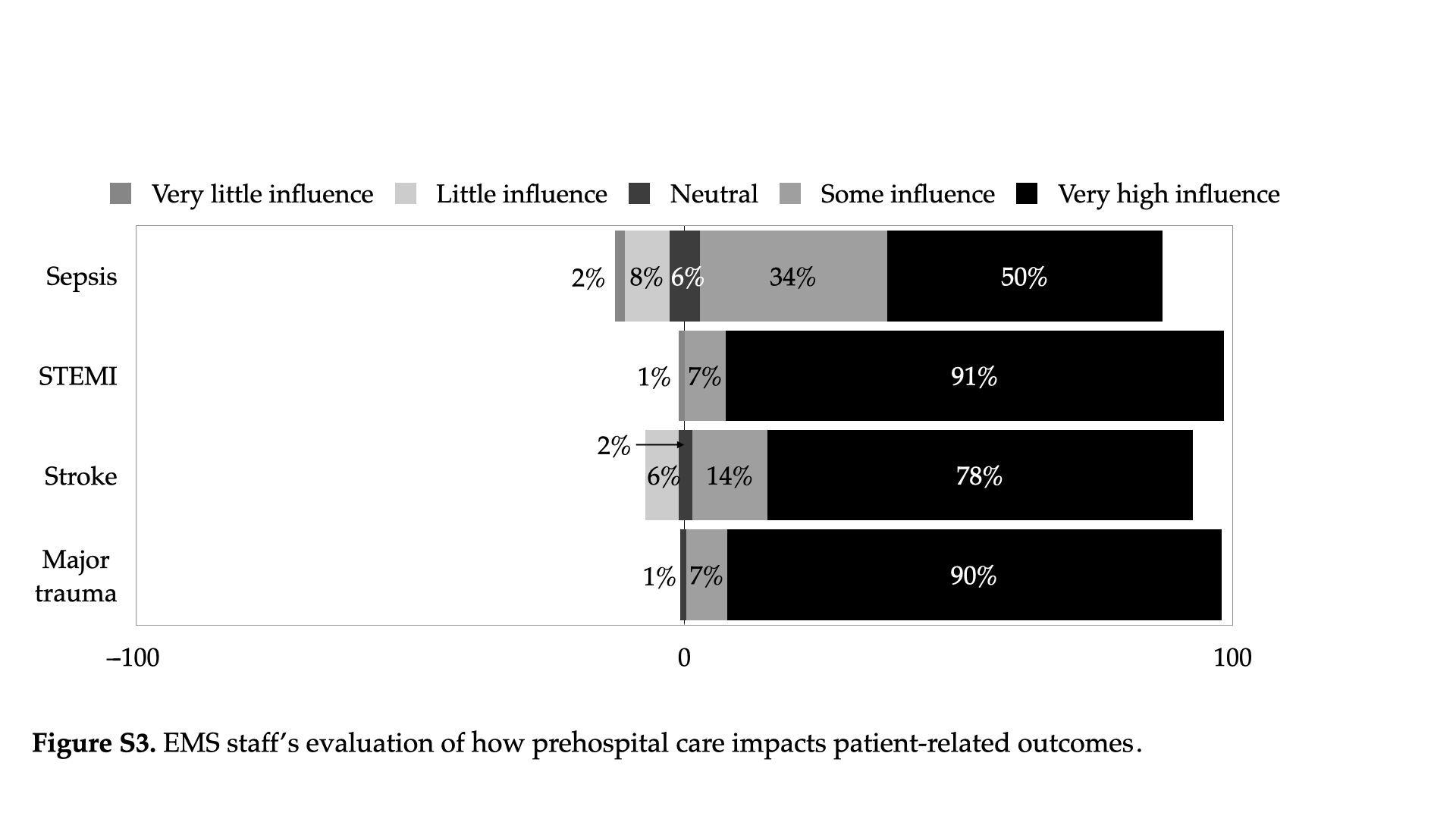

Supplement: Supplementary file 1 [file healthcare-10-01958-s001.zip › Figure S3.jpeg]

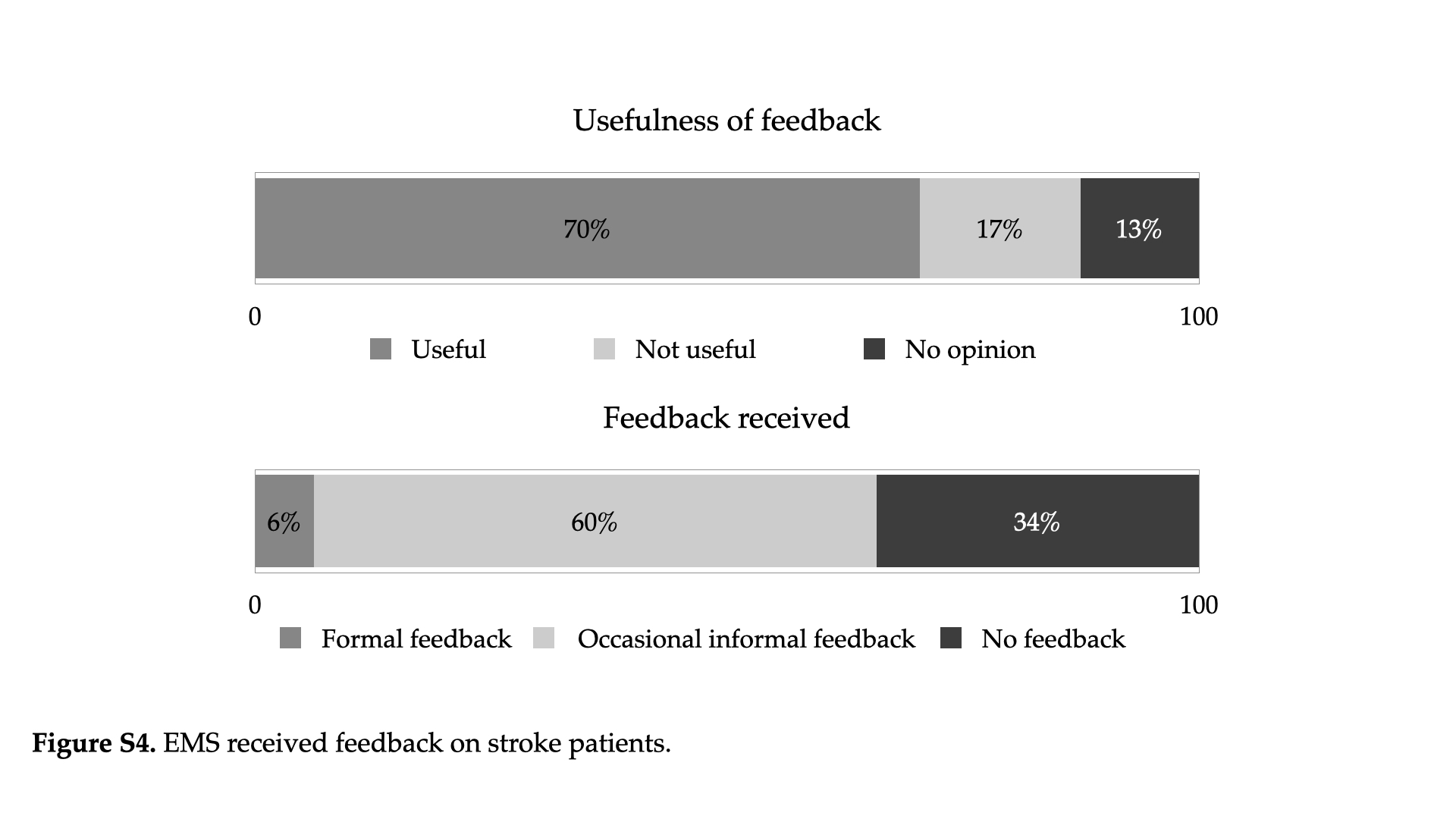

Supplement: Supplementary file 1 [file healthcare-10-01958-s001.zip › Figure S4.jpeg]

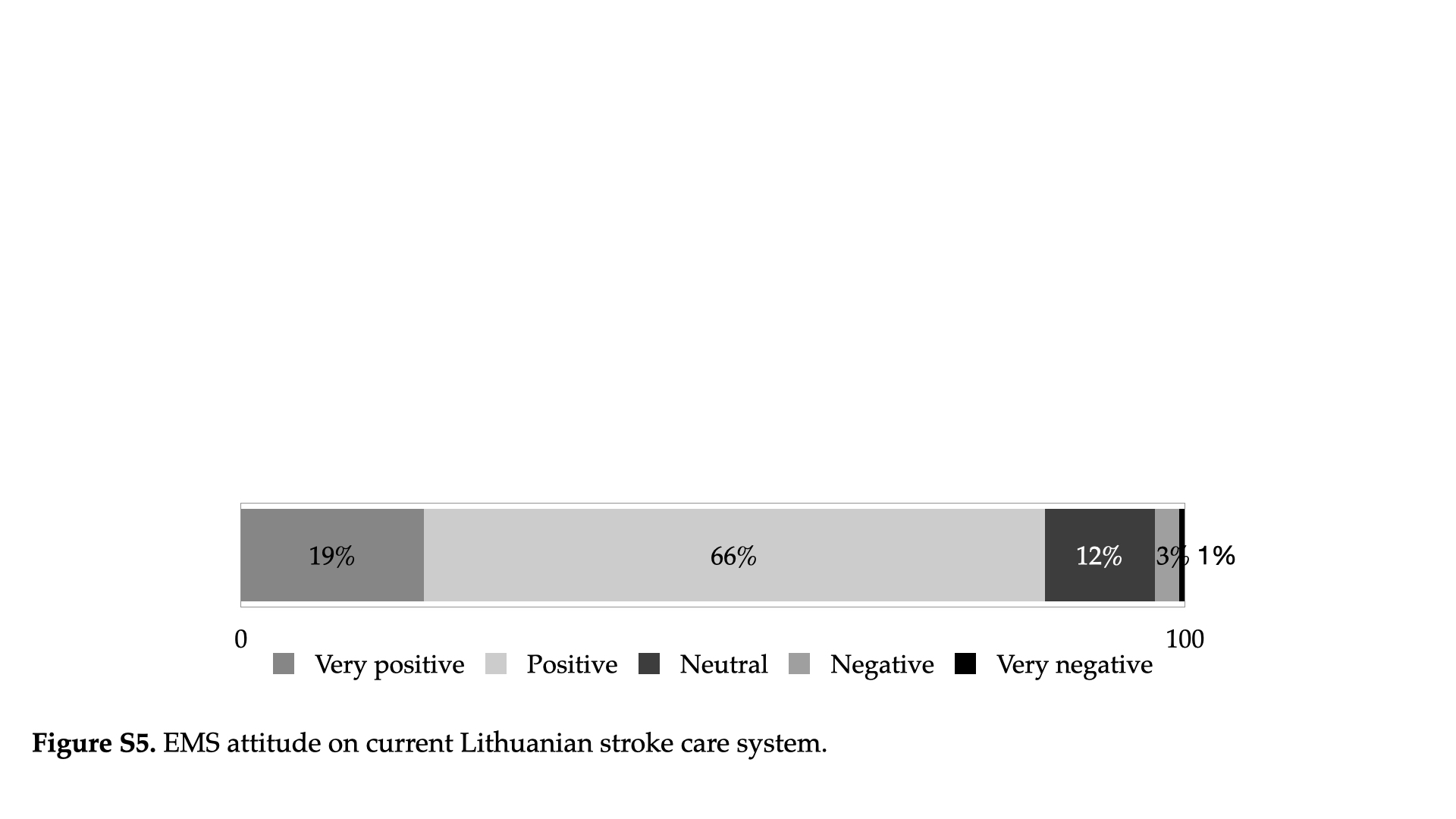

Supplement: Supplementary file 1 [file healthcare-10-01958-s001.zip › Figure S5.jpeg]
